# The Utility of HER2 Overexpression in Prognosis of Gastric Cancer: A Systematic Review and Meta‐Analysis Study

**DOI:** 10.1002/cnr2.70612

**Published:** 2026-06-21

**Authors:** Seyed Morteza Pourfaraji, Alireza Abdollahi, Fatemeh Ojaghi Shirmard, Reza Ghalehtaki, Samaneh Salarvand

**Affiliations:** ^1^ School of Medicine Tehran University of Medical Sciences Tehran Iran; ^2^ Department of Pathology, School of Medicine, IKHC Tehran University of Medical Sciences Tehran Iran; ^3^ Radiation Oncology Research Center, Cancer Research Institute, IKHC Tehran University of Medical Sciences Tehran Iran; ^4^ Department of Radiation Oncology, Cancer Institute, IKHC Tehran University of Medical Sciences Tehran Iran

**Keywords:** gastric cancer, HER2, overexpression, prognosis, survival

## Abstract

**Aim:**

This systematic review and meta‐analysis aimed to clarify the prognostic impact of human epidermal growth factor receptor 2 (HER2) overexpression on overall survival (OS) and progression‐free survival (PFS) in advanced and resectable gastric cancer.

**Methods:**

We systematically searched electronic databases for studies reporting survival outcomes associated with HER2 overexpression in gastric cancer patients. Hazard ratios (HRs) and their corresponding 95% confidence intervals (CIs) were pooled using random‐effects models.

**Results:**

A total of 39 studies with 19 903 gastric cancer patients were included. The pooled rate of HER2 overexpression was estimated at 16% (95% CI, 14% to 18%). Our analysis revealed that HER2 overexpression was significantly associated with worse OS (HR = 1.30, 95% CI 1.12 to 1.51; *p* < 0.01) and PFS (HR = 1.57, 95% CI: 1.09 to 2.26; *p* < 0.01). The association with worse OS was significant across the studies with resectable (HR = 1.31, 95% CI: 1.08 to 1.60; *p* < 0.01) and advanced (HR = 1.25, 95% CI: 1.00 to 1.58; *p* = 0.04) gastric cancer patients. However, the association between HER2 and PFS was only significant in the resectable subgroups (HR = 2.28, 95% CI: 1.09 to 4.74; *p* < 0.01), not in the advanced subgroup (HR = 1.18, 95% CI: 0.93 to 1.51; *p* = 0.16).

**Conclusions:**

HER2 overexpression appears to be associated with poorer outcomes in advanced and resectable gastric cancer. Standardizing diagnostic criteria and integrating HER2 assessment into multimarker panels could enhance prognostic accuracy and guide personalized therapeutic decisions.

## 1 Introduction

Gastric cancer remains a significant global health challenge, ranking as one of the leading causes of cancer‐related mortality worldwide. Despite advances in diagnosis, surgery, chemotherapy, and targeted treatments, the prognosis for gastric cancer, especially in advanced stages, remains poor [1, 2]. Accurate prognostic indicators are thus critical, as they assist clinicians in stratifying patient risk, selecting appropriate therapeutic interventions, and informing patient management strategies [3, 4].

Human epidermal growth factor receptor 2 (HER2) is a transmembrane receptor tyrosine kinase involved in cellular proliferation, differentiation, and survival. Overexpression or amplification of the HER2 gene has been extensively investigated in breast cancer, where it serves as a well‐established prognostic and predictive biomarker. Following these insights, the potential role of HER2 has increasingly been studied in other malignancies, including gastric cancer. In gastric cancer, HER2 positivity has been variably reported, typically ranging from 10% to 30% of cases, and its significance as a prognostic factor remains controversial due to conflicting outcomes observed in previous studies [5, 6, 7].

While some studies have indicated a clear association between HER2 overexpression and worse clinical outcomes in gastric cancer, others have failed to demonstrate significant prognostic differences. This inconsistency may be attributed to variations in study design, diagnostic methods, patient populations, and disease stages examined [8, 9, 10]. As targeted therapies such as trastuzumab have emerged as effective options specifically for HER2‐positive gastric cancer, defining the prognostic role of HER2 expression with greater precision has become clinically essential. This systematic review and meta‐analysis aims to comprehensively evaluate the evidence regarding the prognostic value of HER2 overexpression in gastric cancer.

## 2 Materials and Methods

This systematic review and meta‐analysis adhered to the Preferred Reporting Items for Systematic Reviews and Meta‐Analyses (PRISMA) guidelines.

### 2.1 Systematic Search

A comprehensive search was conducted in electronic databases, including Web of Science, Scopus, and PubMed, encompassing all relevant studies up to January 2025. The search employed Medical Subject Headings (MeSH) and keywords specifically tailored to HER2 testing and gastric cancer, utilizing the following terms: (“hazard” OR “prognos*” OR “survival”) AND (“HER2” OR “Human Epidermal Growth Factor Receptor 2” OR “HER‐2”) AND (“gastric cancer” OR “stomach cancer” OR “gastric carcinoma”). Table S1 demonstrates detailed search strategy.

### 2.2 Inclusion and Eligibility Criteria

Study eligibility was structured using the PICO criteria as follows: Population (P): Patients diagnosed with gastric cancer, inclusive of advanced and resectable cases, irrespective of histological subtype (e.g., adenocarcinoma, signet‐ring cell carcinoma, and others). Intervention (I): Determination of HER2 expression or amplification status (evaluated by immunohistochemistry [IHC] and in situ hybridization [ISH]). Comparison (C): Comparisons between gastric cancer patients with HER2 overexpression/amplification (HER2‐positive) versus those without HER2 overexpression (HER2‐negative). Outcome (O): Prognostic outcomes including overall survival (OS), progression‐free survival (PFS), and HER2 positivity ratio. Both resectable and advanced gastric cancer cohorts were included because HER2 status may have prognostic relevance across different disease stages. Studies were excluded if they involved animal models, case reports, gastric cancers without clear HER2 testing procedures, insufficient prognostic data, or non‐clinical studies (e.g., purely histologic or in vitro analyses). Only studies published after the ToGA trial were included, as this trial established the clinical relevance of HER2 assessment in gastric cancer and was followed by broader use of gastric cancer–specific HER2 diagnostic and scoring criteria.

### 2.3 Data Extraction and Outcome Measurements

Two independent reviewers extracted relevant data using a standardized extraction form, and any discrepancies were resolved through consultation with a third reviewer. Extracted data included authors' names, year of publication, study design, patient demographics, sample sizes, HER2 expression status, follow‐up duration, reported Hazard Ratios (HR) for OS and PFS, and associated confidence intervals (CIs).

### 2.4 Quality Assessments

We evaluated the quality of the included studies using the Newcastle‐Ottawa Scale (NOS) [11]. For cohort studies, the NOS evaluates selection, comparability, and outcome, which are then classified as Good (3–4 stars in selection, 1–2 in comparability, 2–3 in outcome), Fair (2 stars in selection, 1–2 in comparability, 2–3 in outcome), or Poor (0–1 star in selection, 0 in comparability, 0–1 in outcome).

### 2.5 Statistical Analysis and Data Synthesis

The pooled effects for overall survival (OS) and progression‐free survival (PFS) related to HER2 overexpression in gastric cancer were calculated using random‐effects models to accommodate between‐study variability. Both fixed‐effect and random‐effects models were evaluated. Since high heterogeneity was observed in the main analyses, as well as the expected clinical and methodological variability across included studies, the random‐effects model was used as the main model. Fixed‐effect analyses were also performed as sensitivity analyses, and the results are presented in the Supporting Information. Hazard ratios (HRs) were converted to log HRs, and their standard errors were calculated for meta‐analysis. Statistical heterogeneity across studies was quantitatively assessed using the *I* ^2^ statistic, with high heterogeneity predefined as *I* ^2^ > 50%. Forest plots were generated to visually depict pooled estimates and individual study effects. Funnel plots were employed to visually inspect potential publication bias, and the Begg's and Egger's tests were used.

All statistical analyses, including sensitivity and subgroup analyses based on disease stage (advanced vs. resectable gastric cancer), were performed using the meta package in R (R Foundation for Statistical Computing, Vienna, Austria) and RStudio (RStudio Inc., Boston, MA). Sensitivity analyses were specifically conducted to evaluate the stability of the pooled results, assessing the potential influence of individual studies and different subgroups.

## 3 Results

**FIGURE 1:**
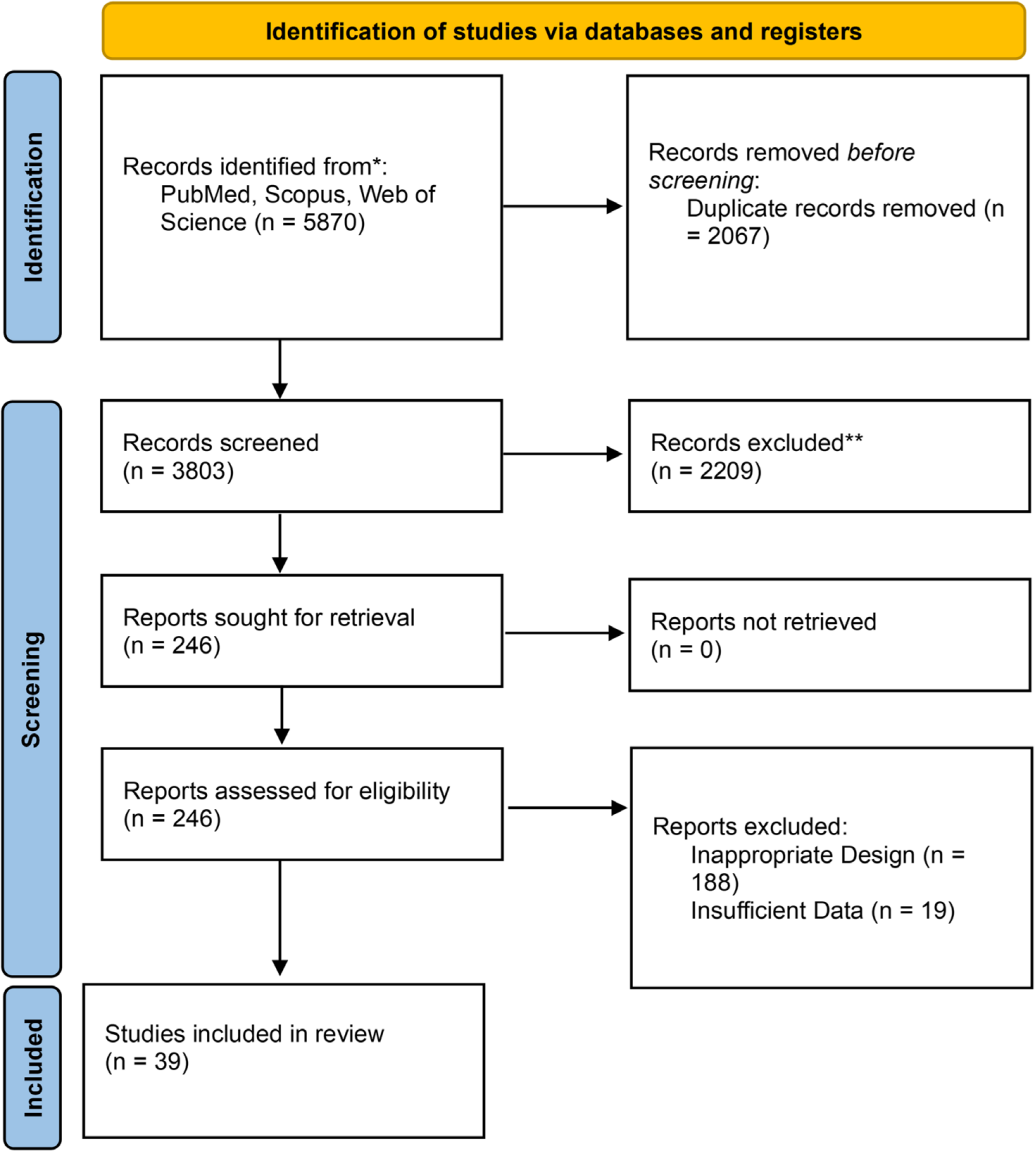
PRISMA flow diagram of the included studies.

Our initial search yielded 5870 articles from PubMed, Scopus, and Web of Science, from which we eliminated 2067 duplicates. After reviewing the titles and abstracts of the remaining 3803 records, we retrieved 246 full‐text articles for further evaluation. Ultimately, 39 studies [12, 13, 14, 15, 16, 17, 18, 19, 20, 21, 22, 23, 24, 25, 26, 27, 28, 29, 30, 31, 32, 33, 34, 35, 36, 37, 38, 39, 40, 41, 42, 43, 44, 45, 46, 47, 48, 49, 50] met our eligibility criteria and were included in the systematic review and meta‐analysis (Figure 1). Detailed characteristics of the included studies are summarized in Table 1. We evaluated the risk of bias of the 39 included cohort studies using the NOS. Of these, 19 studies (48.7%) were rated as good quality (NOS score 7–9), 20 studies (51.3%) as fair quality (NOS score 4–6), and no studies were rated as poor quality (NOS score 0–3) (Table S1).

**TABLE 1:** Detailed Characteristics of the included studies.

Author	Year	Country	Design	Type	HER2 detection		HER2 expression	
					Method	Reference^a^	Positive cases	Sample size
Byeon et al. [12]	2017	Korea	RCS	Advanced	IHC/FISH	Hofmann et al. [51]	32	313
Chen et al. [13]	2021	China	RCS	Resectable	IHC/FISH	ToGA trial [52]	NA	113
Cho et al. [14]	2017	China	RCS	Resectable	IHC/FISH/SISH	Hofmann et al. [51]	32	384
Dai et al. [15]	2013	China	RCS	Advanced	IHC/FISH/HER2:CEP17 Ratio	Hofmann et al. [51] Ruschoff et al. [53]	37	219
Fisher et al. [16]	2014	USA	RCS	Resectable	IHC/FISH	ToGA trial [52]	21	111
Fusco et al. [17]	2013	Italy	RCS	Advanced	IHC/FISH	Hofmann et al. [51] Ruschoff et al. [53]	37	292
Fuse et al. [18]	2016	Japan	RCS	Advanced	IHC/FISH	ToGA trial [52]	26	293
Gao et al. [19]	2023	China	RCS	Resectable	IHC/FISH	Hofmann et al. [51]	378	5622
Gu et al. [20]	2015	China	RCS	Advanced	IHC/FISH	Hofmann et al. [51]	10	92
Guo et al. [21]	2024	China	RCS	Advanced	NA	NA	31	85
Haffner et al. [22]	2021	Germany	PCS	Advanced	IHC/CISH/RT‐qPCR	Ruschoff et al. [53]	77	548
Honma et al. [23]	2014	Japan	RCS	Advanced	IHC/FISH	Hofmann et al. [51]	7	77
Jiang et al. [25]	2015	China	RCS	Resectable	IHC/FISH	ToGA trial [52]	27	227
Jiang et al. [24]	2017	China	PCS	Advanced	IHC/FISH/HER2:CEP17 Ratio	NA	61	290
Junior et al. [26]	2016	Brazil	RCS	Advanced	IHC/FISH	ToGA trial [52]	5	32
Kataoka et al. [27]	2013	Japan	RCS	Resectable	IHC/DISH/HER2:CEP17 Ratio	ToGA trial [52] Ruschoff et al. [53]	25	213
Kurokawa et al. [28]	2015	Japan	RCS	Resectable	IHC/FISH/HER2:CEP17 Ratio	Hofmann et al. [51] ToGA trial [52]	180	1148
Lago et al. [29]	2020	Spain	RCS	Resectable	IHC/CISH/HER2:CEP17 Ratio	Hofmann et al. [51] Ruschoff et al. [53]	14	106
Lee et al. [30]	2017	Korea	RCS	Advanced	IHC/FISH/HER2:CEP17 Ratio	ToGA trial [52]	32	181
Li et al. [31]	2021	China	RCS	Resectable	IHC/FISH/HER2:CEP17 Ratio	Chinese Guidelines [54]	115	1121
Li et al. (i) [32]	2023	China	PCS	Advanced	IHC/ISH	NA	114	238
Li et al. (ii) [33]	2023	China	RCS	Resectable	IHC/ISH	NA	146	1298
Lian et al. [34]	2022	China	PCS	Resectable	IHC/FISH	Wada et al. [55]	13	75
Lin et al. [35]	2024	China	RCS	Resectable	IHC/FISH	ToGA trial [52]	20	83
Lv et al. [36]	2014	China	RCS	Resectable	IHC/FISH/HER2:CEP17 Ratio	Hofmann et al. [51]	24	129
Nagatsuma et al. [37]	2015	Japan	PCS	Advanced	IHC/DISH/HER2:CEP17 Ratio	Hofmann et al. [51]	112	838
Nakayama et al. [38]	2023	Japan	RCS	Resectable	IHC/ISH	Bartley et al. [56]	170	734
Narita et al. [39]	2024	Japan	RCS	Resectable	IHC/ISH	Bartley et al. [56]	120	548
Qiu et al. [40]	2014	China	PCS	Advanced	IHC/FISH/HER2:CEP17 Ratio	Hofmann et al. [51]	98	349
Sheng et al. [41]	2013	China	RCS	Resectable	IHC/FISH/DSISH	Hofmann et al. [51]	91	726
Shi et al. [42]	2017	China	RCS	Resectable	IHC/FISH/HER2:CEP17 Ratio	ToGA trial [52]	39	239
Shitara et al. [43]	2013	Japan	RCS	Advanced	IHC/FISH	ToGA trial [52]	58	364
Tang et al. [44]	2015	China	RCS	Resectable	IHC/FISH	NA	21	121
Terashima et al. [45]	2012	Japan	RCT	Resectable	IHC/DISH/HER2:CEP17 Ratio	Hofmann et al. [51]	58	415
Wei et al. [47]	2020	China	RCS	Resectable	IHC/ISH	NCCN Guidelines	55	195
Wei et al. [46]	2023	China	RCS	Resectable	NA	NA	23	60
Wiegand et al. [48]	2014	Canada	RCS	Resectable	IHC/DISH/HER2:CEP17 Ratio	NA	30	253
Xu et al. [49]	2018	China	RCS	Resectable	IHC/FISH/HER2:CEP17 Ratio	Hofmann et al. [51]	36	280
Xu et al. [50]	2023	China	RCS	Advanced	NA	NA	202	431

Abbreviations: CEP17, chromosome enumeration probe 17; CISH, chromogenic in situ hybridization; DISH, dual‑color in situ hybridization; FISH, fluorescence in situ hybridization; HR, hazard ratio; IHC, immunohistochemistry; L, lower 95% confidence interval; NA, not available; NCCN, National Comprehensive Cancer Network; OS, overall survival; PCS, prospective cohort study; PFS, progression‑free survival; RCS, retrospective cohort study; RT‑qPCR, reverse transcription quantitative polymerase chain reaction; SISH, silver‑enhanced in situ hybridization; ToGA, Trastuzumab for Gastric Cancer trial; U, upper 95% confidence interval.

^a^ Refers to the article cited directly by each included study as the source of the criteria used for HER2 detection.

### 3.1 Overall Survival in Gastric Cancer

**FIGURE 2:**
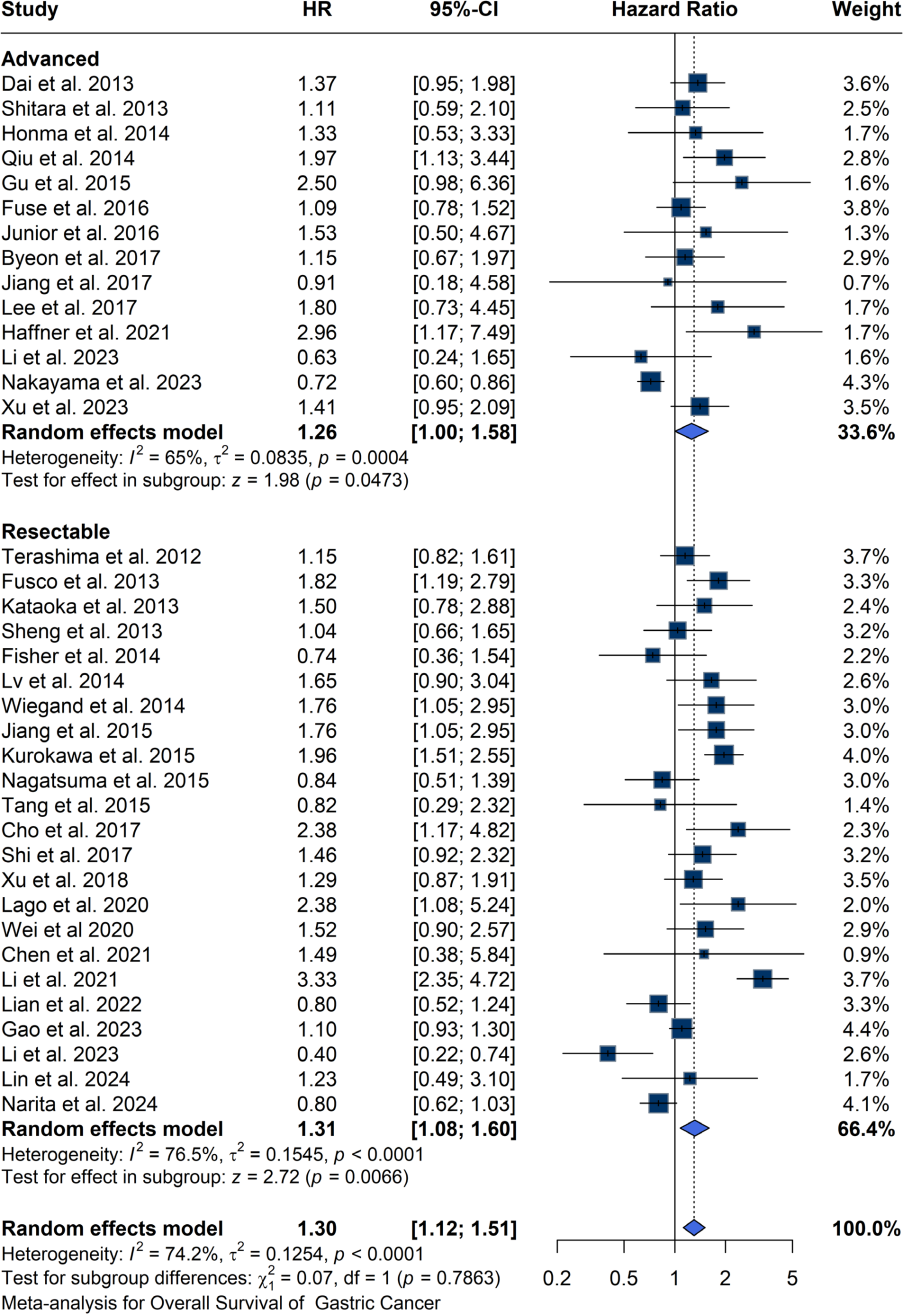
The pooled overall survival of gastric cancer patients; stratified by type of cancer.

The meta‐analysis included 37 studies evaluating the association between HER2 overexpression and overall survival in gastric cancer (Figure 2). The pooled analysis demonstrated that HER2 overexpression was significantly associated with worse overall survival compared to HER2‐negative gastric cancer (HR 1.30, 95% CI 1.12 to 1.51, *p* < 0.01). The results of the fixed‐effect model are represented in Table S3. The between‐study heterogeneity was high (*I* ^2^ = 74.2%, *p* < 0.01). By removing studies one by one, the pooled HR remained consistent, ranging from 1.25 (95% CI: 1.09 to 1.43) by removing Li's study [31] to 1.34 (95% CI: 1.24 to 1.55) by excluding Nakayama's study [38]. The heterogeneity (*I* ^2^) also remained high (72.5% to 74.9%) throughout the sensitivity analysis. This indicates that no single study significantly influenced the overall effect size (Figure S1).

The meta‐analysis of studies assessing HER2 overexpression in advanced gastric cancer included 14 studies. A borderline significant overall effect was identified using random‐effects models (HR = 1.25, 95% CI: 1.00–1.58; *p* = 0.04), suggesting HER2 overexpression may be associated with worse survival in advanced gastric cancer. High heterogeneity (*I* ^2^ = 65%, *p* < 0.01) was observed between included studies. In resectable gastric cancer, the pooled estimate of 23 studies revealed a statistically significant association between HER2 overexpression and overall survival (HR = 1.31, 95% CI: 1.08 to 1.60; *p* < 0.01). High between‐study heterogeneity was observed (*I* ^2^ = 76.5%, *p* < 0.01). No significant between‐group difference in the pooled estimate was found (*p* = 0.78). Figure S2 shows further subgroup analysis stratified by study location. Results revealed significant associations between HER2 positivity and poorer OS in both Asian (HR = 1.25, 95% CI: 1.06 to 1.46; *p* < 0.01) and Western populations (HR = 1.69, 95% CI: 1.23 to 2.32; *p* < 0.01) without a significant between‐group difference (*p* = 0.09). Although heterogeneity remained significant in Asian studies (*I* ^2^ = 75.9%, *p* < 0.01), only moderate, non‐significant between‐study heterogeneity was observed in Western studies (*I* ^2^ = 30.6%, *p* = 0.2).

### 3.2 Progression‐Free Survival in Gastric Cancer

**FIGURE 3:**
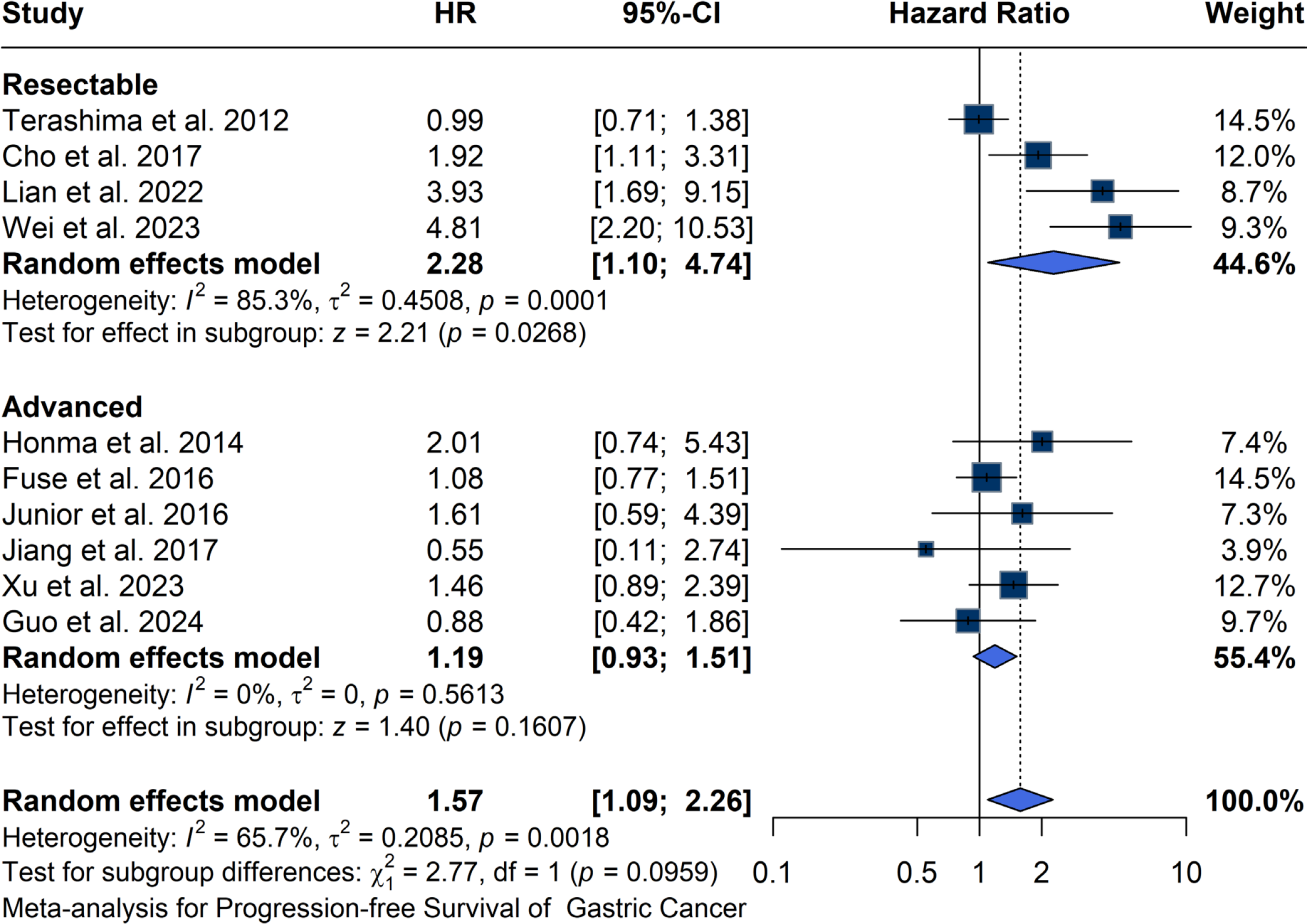
The pooled progression‐free survival of gastric cancer patients; stratified by type of cancer.

A meta‐analysis of 10 studies investigating HER2 status and PFS demonstrated that HER2 overexpression was associated with worse PFS (HR = 1.57, 95% CI: 1.09 to 2.26; *p* < 0.01), while significant heterogeneity was detected (*I* ^2^ = 65.7%, *p* < 0.01) (Figure 3). Sensitivity analysis revealed that the pooled hazard ratio ranged from 1.37 (95% CI: 1.02–1.82) when Wei's study [47] was omitted to 1.70 (95% CI: 1.15–2.52) when Terashima's study [45] was omitted. Removing Wei's study moderated the heterogeneity while removing other studies did not change the between‐study heterogeneity (Figure S3).

The pooled estimate was significant in the subgroup of resectable gastric cancer studies (HR = 2.28, 95% CI: 1.09–4.74; *p* < 0.01), while the association was not statistically significant in the advanced cancer subgroup (HR = 1.18, 95% CI: 0.93 to 1.51; *p* = 0.16). The heterogeneity was higher across the resectable studies (*I* ^2^ = 85.3%) compared to the advanced studies (*I* ^2^ = 0.0%). Furthermore, HER2 positivity was significantly associated with worse PFS in Asian populations (HR = 1.57, 95% CI: 1.06–2.34; *p* = 0.02). Similarly, the single Western study also demonstrated a significant association (Figure S4), although the findings of this subgroup are limited by the inclusion of a single study. No significant between‐subgroup difference was observed (*p* = 0.97). Significant heterogeneity remained among Asian studies (*I* ^2^ = 69.4%, *p* < 0.01), while heterogeneity could not be meaningfully assessed in the Western subgroup due to the limited number of included studies.

### 3.3 Prevalence of HER2 Overexpression in Gastric Cancer

**FIGURE 4:**
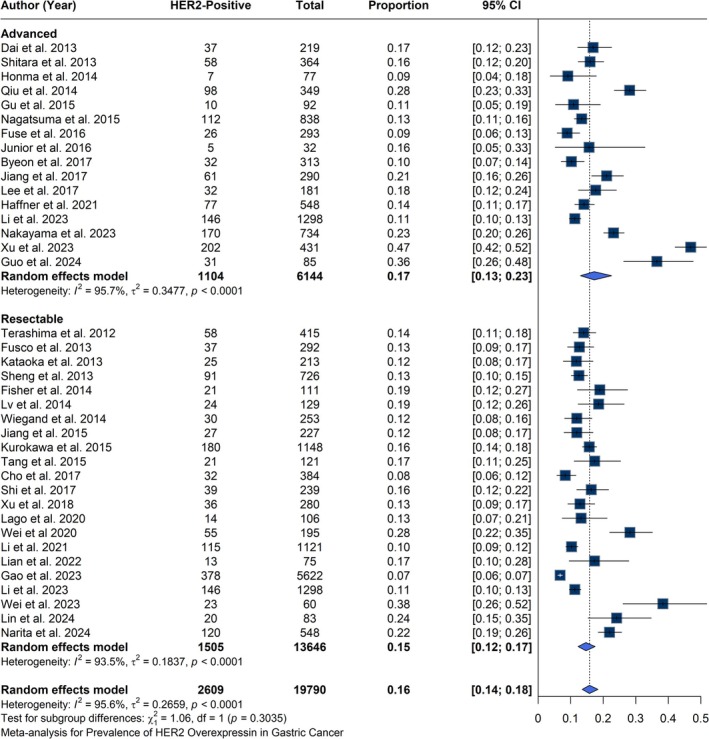
The pooled prevalence of HER2 overexpression in gastric cancer patients; stratified by type of cancer.

The pooled prevalence of HER2 overexpression in gastric cancer was estimated at 16% (95% CI: 14%–18%) across all included studies. A total of 6144 advanced gastric cancer cases were included, and HER2 positivity was identified in 1104 patients, representing 17% incidence (95% CI: 13%–23%). In resectable cancer cases, HER2 overexpression was observed in 1505 of 13 646 cases, yielding a prevalence of 15% (95% CI: 12%–17%). The subgroup analysis showed no statistically significant difference in HER2 positivity rate between Asian (16%) and Western (14%) populations (*p* = 0.14) (Figure 4). However, the analysis revealed that the Western subgroup was homogeneous (*I* ^2^ = 0.0%), whereas the Asian subgroup remained significantly heterogeneous (*I* ^2^ = 96.3%) (Figure S5).

### 3.4 Risk of Publication Bias

**FIGURE 5:**
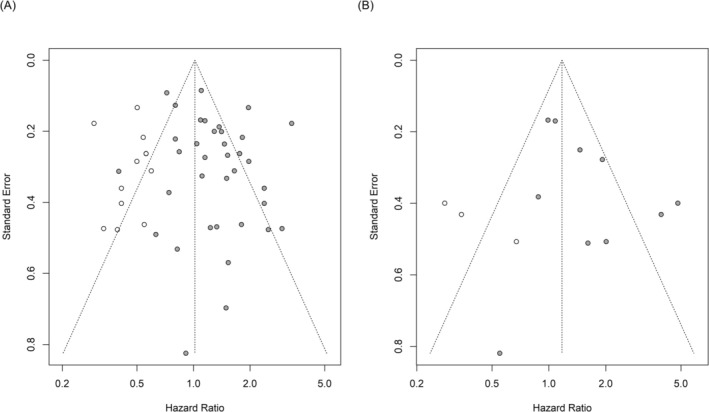
The funnel plots regarding publication bias assessment of (A) overall survival (B) progression‐free Survival.

Visual inspection of the funnel plots revealed a symmetrical distribution of effect sizes, without possible risk of publication bias in OS and PFS analyses (Figure 5). Statistical tests confirmed this observation using Begg's test (*p* = 0.56 and 0.32, respectively) and Egger's test (*p* = 0.45 and 0.39, respectively).

### 3.5 Meta‐Regression Analysis

Meta‐regression analysis was performed using the HER2 positivity ratio and year of publication as covariates to investigate potential sources of heterogeneity. However, neither covariate was significantly associated with the observed between‐study heterogeneity. Detailed results of the meta‐regression analyses are presented in Table S4.

## 4 Discussion

Current prognostic assessment in gastric cancer is primarily based on clinicopathological parameters, including TNM stage, depth of invasion, lymph node involvement, metastatic status, tumor grade, histological subtype, lymphovascular or perineural invasion, resection margin status, and response to perioperative or adjuvant therapy. Although these factors remain critical to risk stratification, they do not completely predict clinical outcomes. Therefore, molecular biomarkers are increasingly suggested to improve the prognostic models and guide treatment selection.

In this comprehensive meta‐analysis, we systematically evaluated both the prevalence of HER2 overexpression and its prognostic significance in patients with gastric cancer. Our findings indicate that the pooled prevalence of HER2 positivity in gastric cancer was approximately 16%, with a 95% confidence interval ranging from 14% to 18%. Since high heterogeneity was observed across studies, it is more suitable to interpret this prevalence as a range rather than a fixed‐point estimate. The prevalence of HER2 overexpression was consistent between subgroups of advanced and resectable gastric tumors, suggesting that the disease stage may not significantly influence HER2 status. Further subgroup analysis suggests that HER2 prevalence may vary by study population/location, with no heterogeneity observed in Western cohorts but high heterogeneity among Asian studies. This variability may reflect differences in tumor location, histological composition, HER2 testing methodology, and population‐specific clinicopathological characteristics. Compared to other malignancies, the rate of HER2 overexpression in gastric cancer is higher than that reported in colorectal cancer (4.1%) and lung cancer (3%). However, it is more closely aligned with the prevalence observed in breast and esophageal cancer (20%), reinforcing the biological and clinical importance of HER2 in gastric malignancies [57, 58, 59, 60].

Our meta‐analysis provides robust evidence of the association between HER2 overexpression and worse OS in both resectable and advanced gastric cancer. However, the impact on PFS was only observed in the subgroup of resectable tumors, which may reflect differences in tumor biology, treatment modalities, or the influence of subsequent therapies in advanced disease. A possible explanation is that HER2 overexpression contributes to earlier recurrence or distant metastasis after surgery, leading to shorter progression‐free intervals in resectable disease. In contrast, in advanced disease, the prognostic significance of HER2 may be diminished by multiple treatment lines, clonal evolution, and variable treatment response.

We did not include the HER2‐positive cases who received any anti‐HER2 drugs (e.g., trastuzumab, pertuzumab) to ensure that the observed prognostic impact is not confounded by the effects of targeted therapy, thereby providing a more precise assessment of HER2's intrinsic prognostic value [52]. Moreover, we only included studies that used Immunohistochemistry (IHC) in combination with in situ hybridization (ISH) methods for detecting HER2 expression that were published after the ToGA trial [52]. Studies that used other methods, such as Cell‐free DNA, circulating tumor DNA, and exosomes, were excluded due to insufficient validation in gastric cancer prognostic studies [56]. These novel methods need to be confirmed by future studies and might help the accuracy of HER2 detection [32]. Despite these strengths, high heterogeneity was observed across studies. Various factors may explain the significant heterogeneity. First, previous studies reported intra‐tumor heterogeneity of HER2 expression in up to 70% of gastric cancer in their cohort. HER2‐positive areas in gastric cancer may be observed just in specific tumor regions, particularly in intestinal‐type and proximal tumors [61, 62]. This may lead to an underestimation of HER2 overexpression in studies that used limited biopsy samples or few sampled tumor sections per case, which may also partly explain the variability in the reported prognostic impact of HER2 across studies. Additionally, recent studies suggest that HER‐2 positivity is often associated with the chromosomal instability (CIN) subtype of gastric cancer, which is characterized by a specific genomic architecture and potentially different clinical behavior. Therefore, HER2 overexpression may represent broader genomic and molecular features of this subtype rather than functioning solely as an independent prognostic biomarker, which may partly explain the observed heterogeneity [63, 64, 65]. Next, the variety in HER2 detection processes across the studies in different timelines, such as using different antibodies or inconsistent adherence to ASCO/CAP scoring (available after 2017), could increase the heterogeneity. Geographical and histological disparities further heighten the heterogeneity [66, 67, 68, 69].

The association between HER2 overexpression and adverse survival outcomes across disease stages may be partly explained by its role as a ligand‐independent receptor tyrosine kinase (TK) that drives oncogenic signaling through heterodimerization [3]. HER2 preferentially forms heterodimers with HER3, creating the most potent signaling complex in the EGFR family. This HER2/HER3 heterodimer can activate phosphoinositide 3‐kinase (PI3K), leading to constitutive activation of the PI3K/AKT/mTOR pathway, which is a critical regulator of cell survival, apoptosis resistance, and metabolic reprogramming [70]. Furthermore, HER2 activates the Ras/Raf/MEK/ERK cascade, which promotes proliferation, epithelial‐mesenchymal transition (EMT), and matrix metalloproteinase (MMP)‐mediated extracellular matrix degradation, potentially facilitating metastatic spread [3, 71, 72]. The translation of these molecular mechanisms into clinical prognosis in gastric cancer appears to be context‐dependent and is influenced by factors including tumor heterogeneity, histological subtype, disease stage, and treatment history. Therefore, HER2‐related signaling may contribute to, rather than fully determine, the more aggressive tumor behavior and worse OS and PFS that are observed in several HER2‐positive gastric cancer cohorts [73, 74, 75].

Based on the observed association between HER2 positivity and adverse survival outcomes, future studies should evaluate HER2 status using standardized testing criteria, adequate tumor sampling, and predefined subgroup analyses according to disease stage, histological subtype, tumor location, and treatment lines.

## 5 Limitations

The current study has several limitations. Significant heterogeneity was observed across included studies, likely due to differences in HER2 detection methods, scoring criteria, and intra‐tumoral heterogeneity. This limits the precision and generalizability of the pooled estimates. Most included studies were retrospective, which may introduce selection bias and limit the ability to control for confounding variables. Additionally, there is the potential for residual confounding related to systemic treatment exposure. Although studies or cohorts in which HER2‐positive patients received anti‐HER2 therapy were excluded whenever this information was available, treatment details were not uniformly reported across all included studies. Another limitation is that HER2 status in gastric cancer seems to be a predictive factor, rather than only prognostic, especially due to the use of trastuzumab and newer HER2‐targeted therapies. Therefore, the observed survival associations may partly reflect differences in treatment era, access to HER2‐directed therapy, various treatment lines, and residual confounding, rather than tumor aggressiveness itself. Furthermore, studies using novel HER2 detection techniques (e.g., circulating tumor DNA, exosomes) were excluded due to limited number and unknown validity, potentially underestimating the true prevalence and prognostic value of HER2 in gastric cancer. Although funnel plot analysis and Begg's test did not reveal significant publication bias, the possibility of unpublished studies with non‐significant associations cannot be entirely excluded.

## 6 Conclusions

This systematic review and meta‐analysis highlight the prognostic significance of HER2 overexpression in gastric cancer, demonstrating that HER2 positivity is potentially associated with poorer OS and PFS. The implementation of standardized HER2 testing protocols and the integration of HER2 status into multimarker panels could further refine risk stratification and guide personalized treatment decisions. Further prospective, large‐scale studies are required to validate these conclusions and fully elucidate HER2's role in guiding clinical decisions and therapeutic strategies in gastric cancer management.

## Author Contributions

**Reza Ghalehtaki:** data curation, supervision, resources, writing – review and editing, conceptualization, writing – original draft. **Seyed Morteza Pourfaraji:** conceptualization, investigation, writing – original draft, writing – review and editing, visualization, formal analysis, data curation, validation, software. **Fatemeh Ojaghi Shirmard:** writing – original draft, writing – review and editing, data curation. **Alireza Abdollahi:** conceptualization, writing – original draft, methodology, data curation, software, supervision, writing – review and editing, resources. **Samaneh Salarvand:** writing – review and editing, validation, funding acquisition, project administration, resources, supervision, data curation, conceptualization, writing – original draft.

## Funding

The authors have nothing to report.

## Conflicts of Interest

The authors declare no conflicts of interest.

## Supporting information


**Table S1:** Detailed search strategy for each library.
**Table S2:** Quality assessment of included articles (Cohorts) bases on the Newcastle—Ottawa Scale.
**Table S3:** The findings of sensitivity analysis using fixed‐effect model.
**Table S4:** The findings of meta‐regression analysis.
**Figure S1:** The Forest plot displays the results of a leave‐one‐out sensitivity analysis for the association between HER2 overexpression and overall survival (OS) in gastric cancer.
**Figure S2:** The pooled Overall Survival of Gastric Cancer Patients; Stratified by Population of Study.
**Figure S3:** The Forest plot displays the results of a leave‐one‐out sensitivity analysis for the association between HER2 overexpression and progression‐free survival (PFS) in gastric cancer.
**Figure S4:** The pooled Progression Free Survival of Gastric Cancer Patients; Stratified by Population of Study.
**Figure S5:** The pooled Prevalence of HER2 Overexpression of Gastric Cancer Patients; Stratified by Population of Study.

## Data Availability

The data that support the findings of this study are available on request from the corresponding author. The data are not publicly available due to privacy or ethical restrictions.
