# Supplementary material for: The Utility of HER2 Overexpression in Prognosis of Gastric Cancer: A Systematic Review and Meta‐Analysis Study
Source: Cancer Rep (Hoboken). 2026 Jun 21;9(6):e70612. doi: 10.1002/cnr2.70612 (PMC13283906; doi:10.1002/cnr2.70612)
Supplement: Supplementary file 1 — Table S1: Detailed search strategy for each library. Table S2: Quality assessment of included articles (Cohorts) bases on the Newcastle—Ottawa Scale. Table S3: The findings of sensitivity analysis using fixed‐effect model. Table S4: The findings of meta‐regression analysis. Figure S1: The Forest plot displays the results of a leave‐one‐out sensitivity analysis for the association between HER2 overexpression and overall survival (OS) in gastric cancer. Figure S2: The pooled Overall Survival of Gastric Cancer Patients; Stratified by Population of Study. Figure S3: The Forest plot displays the results of a leave‐one‐out sensitivity analysis for the association between HER2 overexpression and progression‐free survival (PFS) in gastric cancer. Figure S4: The pooled Progression Free Survival of Gastric Cancer Patients; Stratified by Population of Study. Figure S5: The pooled Prevalence of HER2 Overexpression of Gastric Cancer Patients; Stratified by Population of Study. [file CNR2-9-e70612-s001.docx]

**The Utility of HER2 Overexpression in Prognosis of Gastric Cancer: A Systematic Review and Meta-Analysis Study**

Seyed Morteza Pourfaraji^1^, Alireza Abdollahi^2^, Fatemeh Ojaghi Shirmard^1^, Reza Ghalehtaki^3,4^, Samaneh Salarvand^2*^

^1^School of Medicine, Tehran University of Medical Sciences, Tehran, Iran

^2^Department of Pathology, School of Medicine, IKHC, Teheran University of Medical Sciences, Tehran, Iran

^3^Radiation Oncology Research Center, Cancer Research Institute, IKHC, Tehran University of Medical Sciences, Tehran, Iran

^4^Department of Radiation Oncology, Cancer Institute, IKHC, Tehran University of Medical Sciences, Tehran, Iran

***Correspondence Author**

Samaneh Salarvand

sm.salarvand@gmail.com

Department of Pathology, School of Medicine, IKHC, Teheran University of Medical Sciences, Tehran, Iran

**Supplemental Table 1.** Detailed search strategy for each library

**PubMed**

|  | Query | Numbers |
| --- | --- | --- |
| #1 | ("hazard"[Title/Abstract] OR "prognos*"[Title/Abstract] OR "survival"[Title/Abstract] OR "overall survival"[Title/Abstract] OR "progression-free survival"[Title/Abstract] OR "disease-free survival"[Title/Abstract]) | 2,210,425 |
| #2 | ("HER2"[Title/Abstract] OR "HER-2"[Title/Abstract] OR "ERBB2"[Title/Abstract] OR "Human Epidermal Growth Factor Receptor 2"[Title/Abstract]) | 63,192 |
| #3 | ("gastric cancer"[Title/Abstract] OR "stomach cancer"[Title/Abstract] OR "gastric carcinoma"[Title/Abstract] OR "stomach carcinoma"[Title/Abstract] OR "gastric neoplasm*"[Title/Abstract] OR "stomach neoplasm*"[Title/Abstract] OR "Stomach Neoplasms"[MeSH Terms]) | 137,941 |
| #4 | #1 AND #2 AND #3 | **1,791** |

**Scopus**

|  | **Query** | **Numbers** |
| --- | --- | --- |
| #1 | TITLE-ABS-KEY(hazard OR prognos* OR survival OR "overall survival" OR "progression-free survival" OR "disease-free survival") | 2,461,455 |
| #2 | TITLE-ABS-KEY(HER2 OR "HER-2" OR ERBB2 OR "Human Epidermal Growth Factor Receptor 2") | 83,104 |
| #3 | TITLE-ABS-KEY("gastric cancer" OR "stomach cancer" OR "gastric carcinoma" OR "stomach carcinoma" OR "gastric neoplasm*" OR "stomach neoplasm*") | 177,902 |
| #4 | #1 AND #2 AND #3 | **2,440** |

**Web of Science**

|  | **Query** | **Numbers** |
| --- | --- | --- |
| #1 | TS=(hazard OR prognos* OR survival OR "overall survival" OR "progression-free survival" OR "disease-free survival") | 1,492,129 |
| #2 | TS=(HER2 OR "HER-2" OR ERBB2 OR "Human Epidermal Growth Factor Receptor 2") | 36,142 |
| #3 | TS=("gastric cancer" OR "stomach cancer" OR "gastric carcinoma" OR "stomach carcinoma" OR "gastric neoplasm*" OR "stomach neoplasm*") | 166,683 |
| #4 | #1 AND #2 AND #3 | **1,639** |

**Supplemental Table 2.** Quality assessment of included articles (Cohorts) bases on the Newcastle – Ottawa Scale

| **Study**  **(Year)** | **Selection** | | | | **Comparability** | | **Outcome** | | | **Overall**  **Score^*^** |
| --- | --- | --- | --- | --- | --- | --- | --- | --- | --- | --- |
|  | **Representation** | **Non-Exposed** | **Ascertainment** | **Demonstration** | **Important** | **other** | **Outcome** | **Follow-up length** | **Follow-up adequacy** |  |
| Byeon et al. 2017 | 1 | 0 | 1 | 1 | 1 | 1 | 1 | 1 | 1 | 8 |
| Chen et al. 2021 | 1 | 0 | 1 | 1 | 1 | 0 | 1 | 1 | 0 | 6 |
| Cho et al. 2017 | 1 | 1 | 1 | 0 | 1 | 0 | 1 | 1 | 1 | 7 |
| Dai et al. 2013 | 1 | 1 | 1 | 1 | 1 | 0 | 1 | 1 | 1 | 8 |
| Fisher et al. 2014 | 1 | 1 | 1 | 0 | 1 | 1 | 1 | 1 | 1 | 8 |
| Fusco et al. 2013 | 1 | 1 | 1 | 0 | 1 | 0 | 1 | 1 | 1 | 7 |
| Fuse et al. 2016 | 1 | 1 | 1 | 0 | 1 | 0 | 1 | 1 | 1 | 7 |
| Gao et al. 2023 | 1 | 0 | 1 | 1 | 1 | 0 | 1 | 1 | 0 | 6 |
| Gu et al. 2014 | 0 | 1 | 1 | 1 | 1 | 0 | 1 | 1 | 0 | 6 |
| Guo et al. 2024 | 0 | 1 | 1 | 1 | 1 | 0 | 1 | 1 | 0 | 6 |
| Haffner et al. 2021 | 1 | 1 | 1 | 1 | 1 | 1 | 1 | 1 | 1 | 9 |
| Honma et al. 2014 | 1 | 1 | 1 | 0 | 0 | 1 | 1 | 1 | 0 | 6 |
| Jiang et al. 2016 | 1 | 1 | 1 | 0 | 0 | 1 | 1 | 1 | 0 | 6 |
| Jiang et al. 2015 | 1 | 1 | 1 | 0 | 1 | 0 | 1 | 1 | 0 | 6 |
| Junior et al. 2016 | 1 | 1 | 1 | 0 | 0 | 1 | 1 | 1 | 0 | 6 |
| Kataoka et al. 2012 | 1 | 1 | 1 | 1 | 1 | 0 | 1 | 1 | 0 | 7 |
| Kurokawa et al. 2014 | 1 | 1 | 1 | 1 | 1 | 1 | 1 | 1 | 0 | 8 |
| Lago et al. 2017 | 0 | 1 | 1 | 1 | 1 | 0 | 1 | 1 | 0 | 6 |
| Lee et al. 2017 | 0 | 1 | 1 | 0 | 0 | 1 | 1 | 1 | 0 | 5 |
| Li et al. 2020 | 1 | 1 | 1 | 1 | 1 | 1 | 1 | 1 | 1 | 9 |
| Li et al. (i). 2024 | 0 | 1 | 1 | 0 | 1 | 1 | 0 | 1 | 1 | 6 |
| Li et al. (ii). 2023 | 1 | 1 | 1 | 0 | 1 | 0 | 1 | 1 | 0 | 6 |
| Lian et al. 2022 | 0 | 1 | 1 | 0 | 1 | 1 | 1 | 1 | 0 | 6 |
| Lin et al. 2024 | 0 | 1 | 1 | 0 | 1 | 0 | 1 | 1 | 0 | 5 |
| Lv et al. 2014 | 0 | 1 | 1 | 0 | 1 | 0 | 1 | 1 | 0 | 5 |
| Nagatsuma et al. 2014 | 1 | 1 | 1 | 0 | 0 | 1 | 1 | 1 | 0 | 6 |
| Nakayama et al. 2023 | 1 | 1 | 1 | 0 | 1 | 1 | 1 | 1 | 0 | 7 |
| Narita et al. 2024 | 1 | 1 | 1 | 0 | 1 | 1 | 1 | 1 | 0 | 7 |
| Qiu et al.2014 | 1 | 1 | 1 | 0 | 0 | 1 | 1 | 1 | 0 | 6 |
| Sheng et al.2013 | 1 | 1 | 1 | 0 | 1 | 0 | 1 | 1 | 0 | 6 |
| Shi et al.2017 | 1 | 1 | 1 | 1 | 1 | 0 | 1 | 1 | 0 | 7 |
| Shitara et al.2013 | 1 | 1 | 1 | 0 | 1 | 1 | 1 | 1 | 0 | 7 |
| Tang et al.2014 | 1 | 1 | 1 | 0 | 0 | 1 | 1 | 1 | 0 | 6 |
| Terashima et al.2012 | 1 | 1 | 1 | 1 | 1 | 1 | 1 | 1 | 1 | 9 |
| Wei et al.2023 | 0 | 1 | 1 | 0 | 1 | 0 | 1 | 1 | 0 | 5 |
| Wei et al.2020 | 1 | 1 | 1 | 0 | 1 | 1 | 1 | 1 | 0 | 7 |
| Wiegand et al.2014 | 1 | 1 | 1 | 1 | 1 | 1 | 1 | 1 | 1 | 9 |
| Xu et al.2018 | 1 | 1 | 1 | 0 | 1 | 1 | 1 | 1 | 0 | 7 |
| Xu et al.2023 | 1 | 1 | 1 | 0 | 1 | 1 | 1 | 1 | 0 | 7 |
| ^*^Good (3–4 stars in selection, 1-2 in comparability, 2-3 in outcome), Fair (2 stars in selection, 1-2 in comparability, 2-3 in outcome), or Poor (0–1 star in selection, 0 in comparability, 0–1 in outcome) | | | | | | | | | | |

| **Supplemental Table 3.** The findings of sensitivity analysis using fixed-effect model | | | | | |
| --- | --- | --- | --- | --- | --- |
| Main Variable | Pooled estimate  HR (95% CI) | P value | Heterogeneity (I^2^) | P value |  |
| Overall Survival | 1.183 (1.104 to 1.267) | < 0.01 | 74.2% | < 0.01 |  |
| Progression free survival | 1.336 (1.122 to 1.590) | < 0.01 | 65.7% | < 0.01 |  |
|  |  |  |  |  |  |

| **Supplemental Table 4.** The findings of meta-regression analysis | | | | |
| --- | --- | --- | --- | --- |
| Main Variable | Predictor variable | Amount of heterogeneity accounted for | Residual heterogeneity (I^2^) | Test of moderator (P value) |
| Overall Survival | Year of publication | 10.18% | 69.09% | 0.09 |
|  | HER2 positivity ratio | 0.0% | 72.81% | 0.71 |
| Progression free survival | Year of publication | 11.05% | 65.7% | 0.22 |
|  | HER2 positivity ratio | 0.0% | 73.04% | 0.72 |


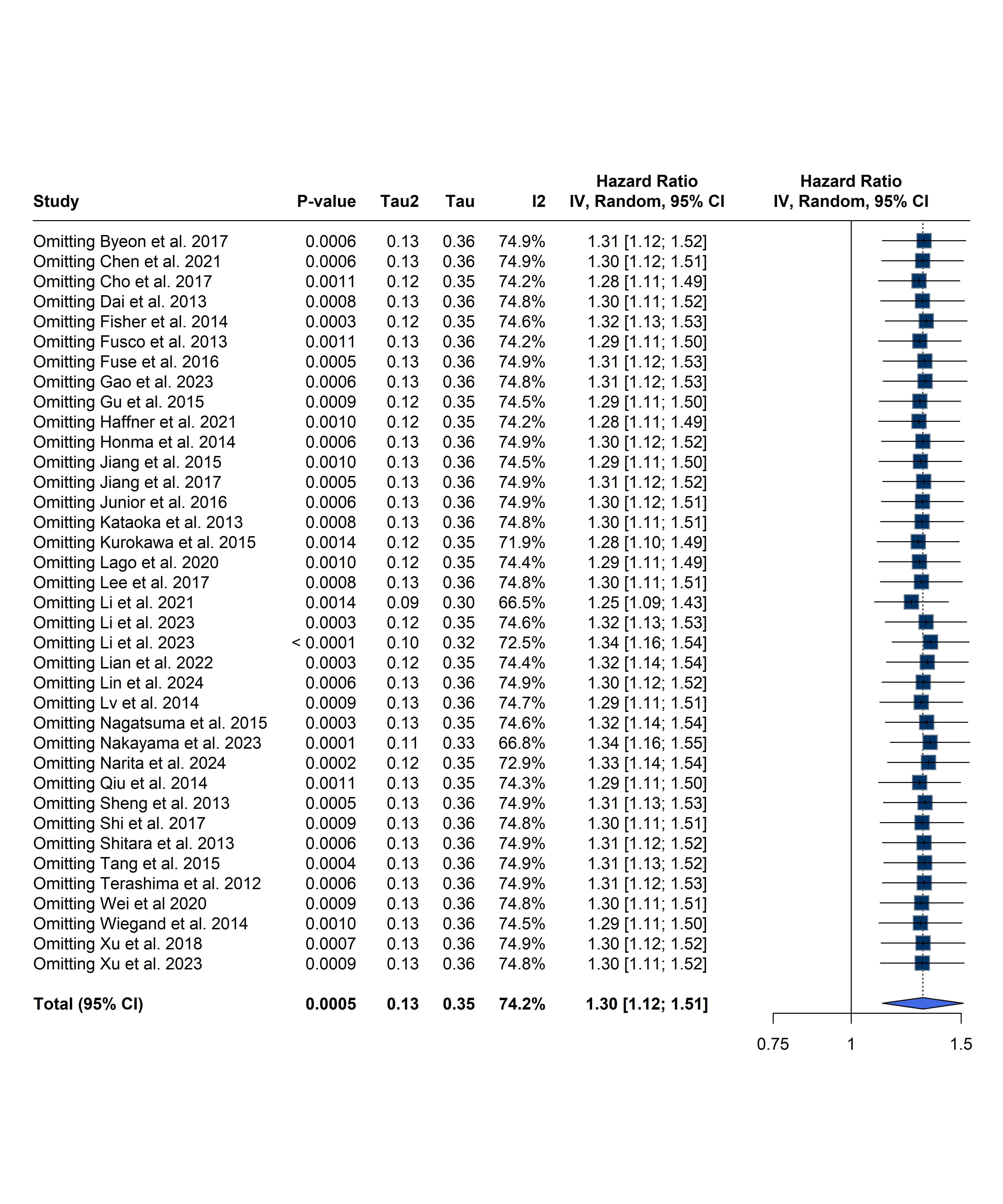


**Supplemental Figure 1.** The Forest plot displays the results of a leave-one-out sensitivity analysis for the association between HER2 overexpression and overall survival (OS) in gastric cancer

**Supplemental Figure 2.** The pooled Overall Survival of Gastric Cancer Patients; Stratified by
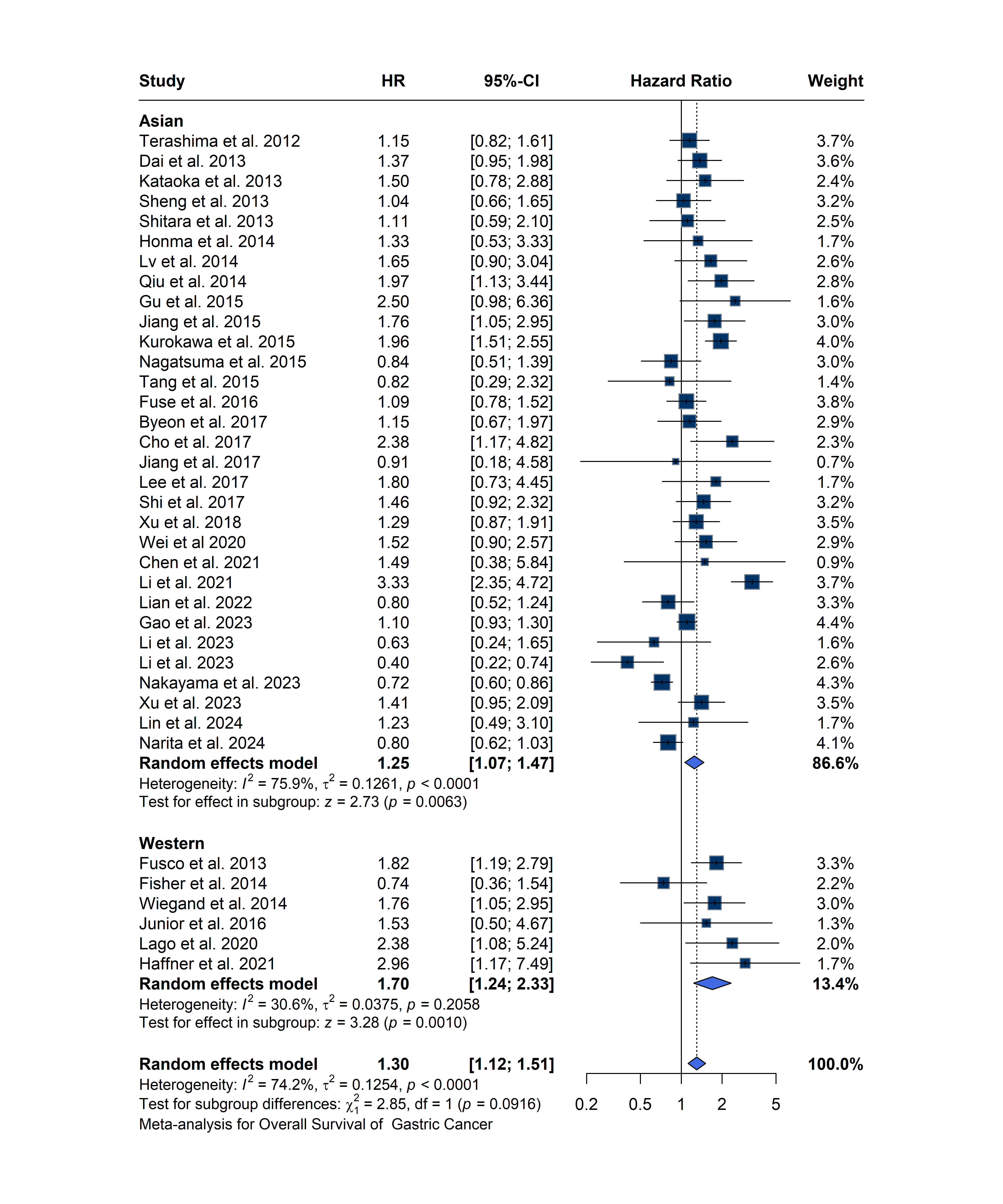
Population of Study


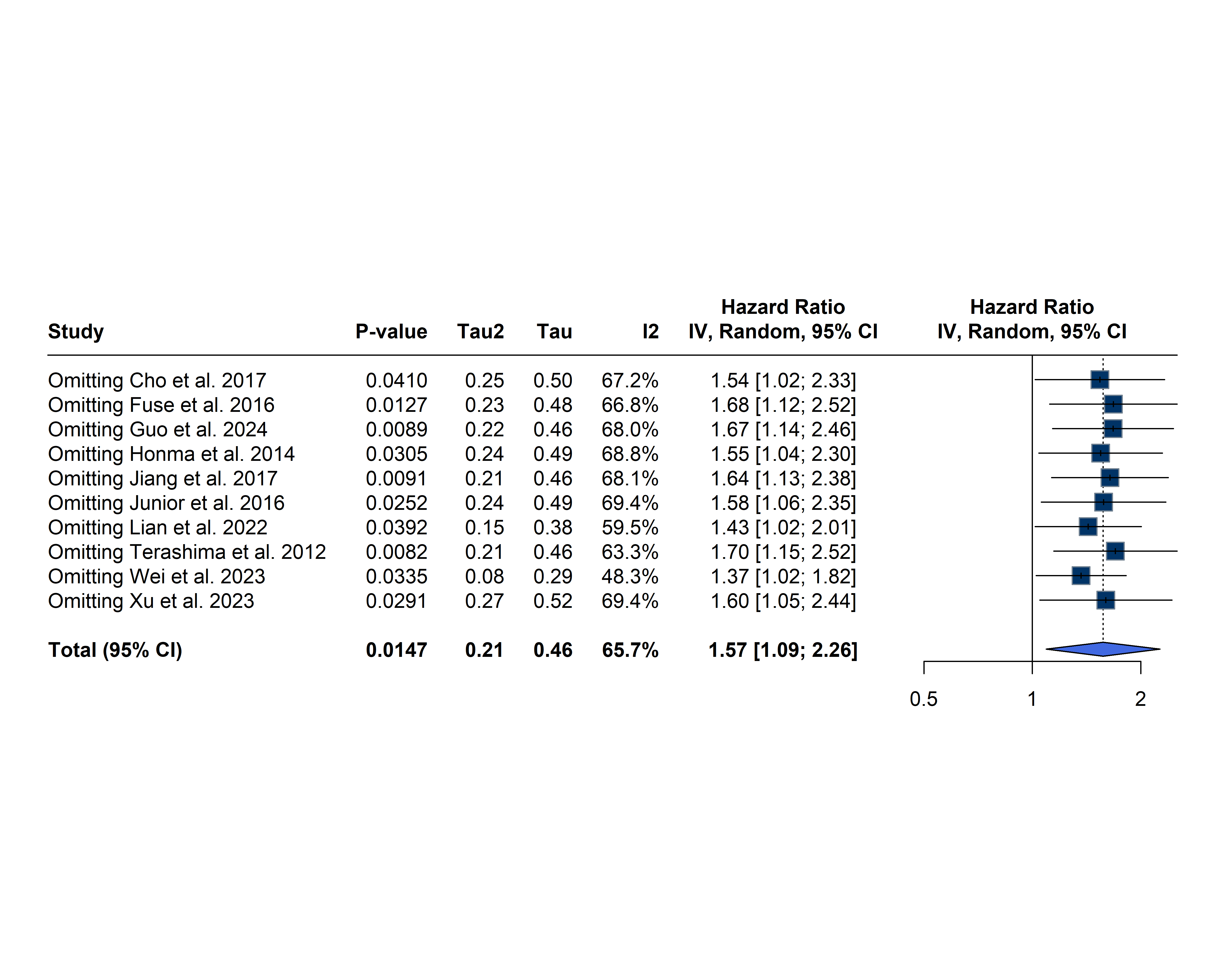


**Supplemental Figure 3.** The Forest plot displays the results of a leave-one-out sensitivity analysis for the association between HER2 overexpression and progression-free survival (PFS) in gastric cancer

**Supplemental Figure 4.** The pooled Progression Free Survival of Gastric Cancer Patients; Stratified by
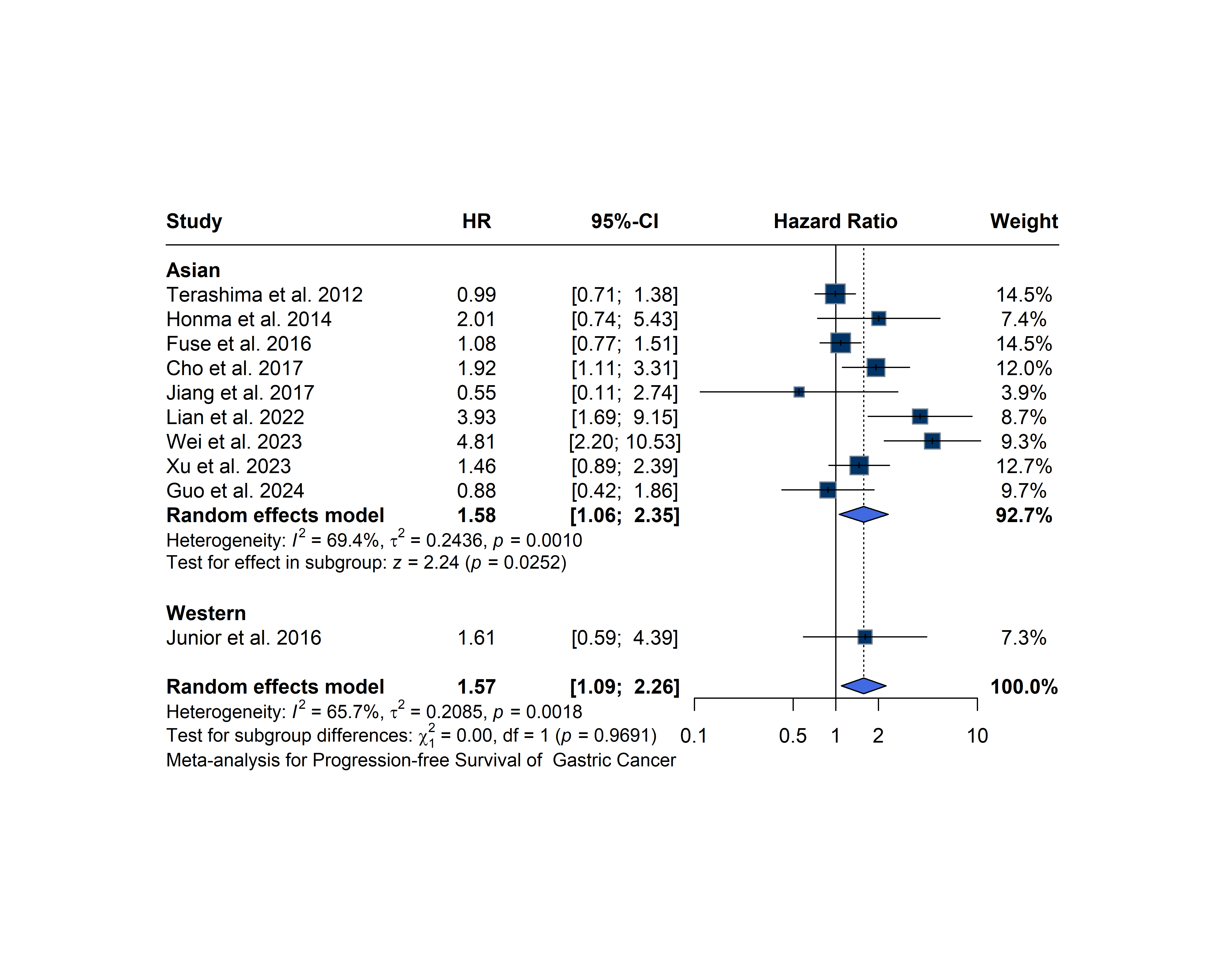
Population of Study


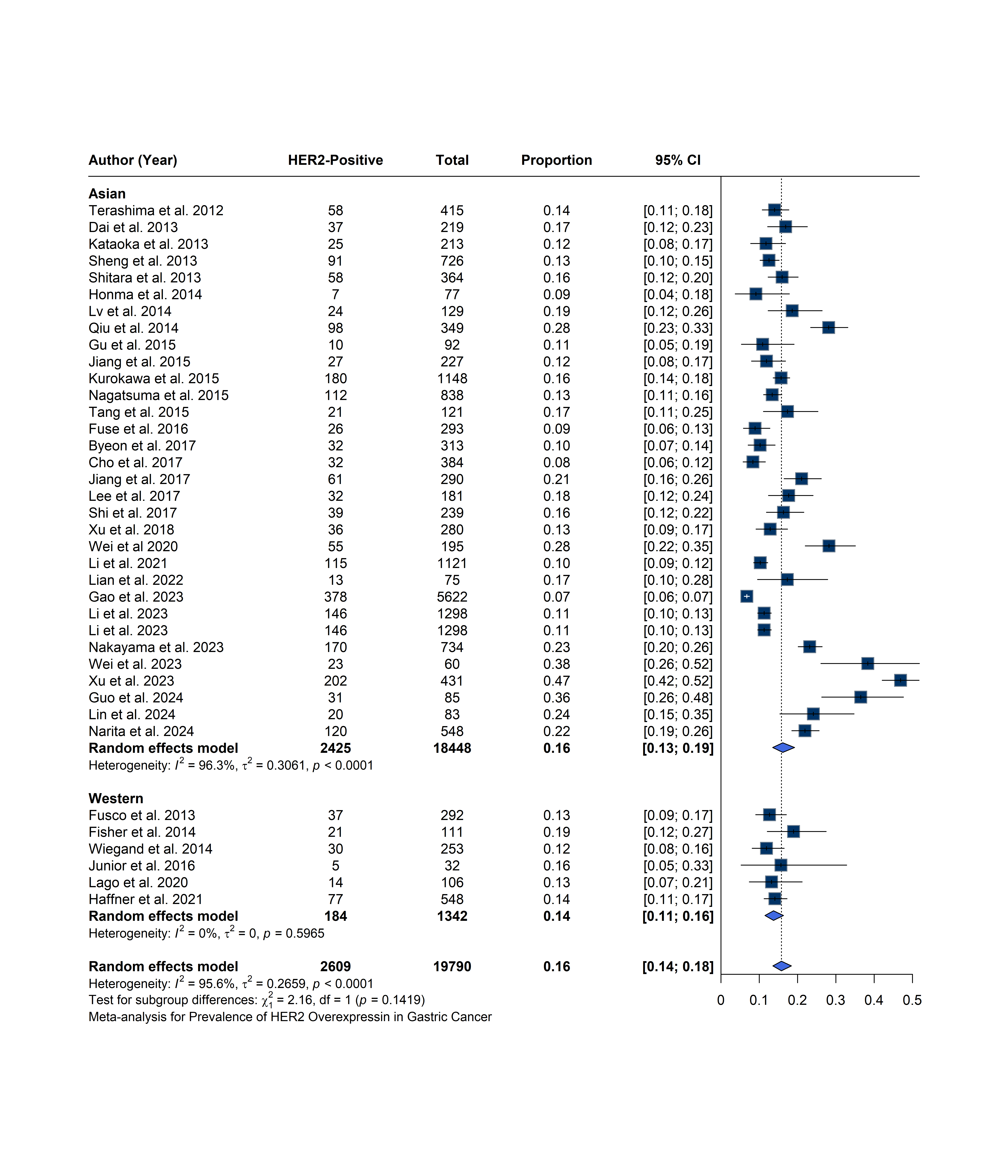


**Supplemental Figure 5.** The pooled Prevalence of HER2 Overexpression of Gastric Cancer Patients; Stratified by Population of Study
